# PD‐L1 and PD1 expression in post‐transplantation lymphoproliferative disease (PTLD) of childhood and adolescence: An inter‐ and intra‐individual descriptive study covering the whole spectrum of PTLD categories

**DOI:** 10.1002/cam4.2394

**Published:** 2019-07-03

**Authors:** Ana‐Iris Schiefer, Elisabeth Salzer, Anna Füreder, Zsolt Szepfalusi, Thomas Müller‐Sacherer, Wolf‐Dietrich Huber, Ina Michel‐Behnke, Anita Lawitschka, Herbert Pichler, Georg Mann, Caroline Hutter, Ingrid Simonitsch‐Klupp, Andishe Attarbaschi

**Affiliations:** ^1^ Department of Pathology Medical University of Vienna Vienna Austria; ^2^ Department of Pediatric Hematology and Oncology St. Anna Children's Hospital Vienna Austria; ^3^ Department of Pediatrics and Adolescent Medicine Medical University of Vienna Vienna Austria; ^4^ CeMM Research Center for Molecular Medicine of the Austrian Academy of Sciences Vienna Austria; ^5^ Ludwig Boltzmann Institute for Rare and Undiagnosed Diseases Vienna Austria; ^6^ Department of Pediatrics and Adolescent Medicine Division of Pediatric Cardiology, Pediatric Heart Center Medical University of Vienna Vienna Austria

**Keywords:** expression, PD1, PD‐L1, post‐transplantation lymphoproliferative disease (PTLD), PTLD category

## Abstract

Therapy of children with post‐transplantation lymphoproliferative disorder (PTLD) after hematopoietic stem cell (HSCT) and solid organ transplantation (SOT) can be challenging. In this retrospective study, we investigated PD‐L1 and PD1 expression in all PTLD categories of childhood and adolescence to see whether checkpoint inhibition with PD‐L1/PD1 inhibitors may serve as a therapy option. We included 21 patients aged 19 years or younger (at date of transplant) with PTLD following SOT or HSCT having adequate tumor samples available (n = 29). Using immunohistochemistry, we evaluated PD‐L1/PD1 expression on both tumor cells and cells of the microenvironment in all samples. Availability of consecutively matched tumor samples during 6 of 21 patients' disease courses also allowed an intra‐individual assessment of PD‐L1/PD1 expression. We observed lower PD‐L1 and higher PD1 expression in non‐destructive lesions, and higher PD‐L1 and lower PD1 expression in polymorphic and, in particular, in monomorphic PTLD, mostly diffuse large B‐cell lymphomas (DLBCL, n = 10/21). The amount of PD‐L1‐ and PD1‐positive cells changed in the opposite way in sequential biopsies of the same individual correlating well with the PTLD category. This is the first comprehensive pediatric study assessing PD‐L1 and PD1 expression on tumor cells and in the microenvironment of PTLD including not only monomorphic, but also non‐destructive early lesions. PD‐L1 expression of the tumor cells inversely correlated with PD1 expression in surrounding tissues, with the highest expression in DLBCL. Since PTLD can be therapeutically challenging, our results indicate a potential efficacy of checkpoint inhibitors if standard immune‐ and/or chemotherapy fail or are impossible. We therefore recommend routine staining of PD‐L1 and PD1 in all PTLD categories.

## 1 INTRODUCTION

Post‐transplantation lymphoproliferative disorder (PTLD) comprises a variety of clinical and pathologic entities that may develop in the setting of decreased T‐cell function and disturbed immune surveillance after hematopoietic stem cell transplantation (HSCT) and solid organ transplantation (SOT).^1^ With the increasing number of SOT performed and improved long‐term post‐transplant survival, PTLD has become the most common malignancy following SOT during childhood and adolescence.^2^, ^3^ It is also significantly contributing to the number of non‐Hodgkin's lymphoma [NHL, specifically, diffuse large B‐cell lymphoma (DLBCL) and Burkitt's lymphoma (BL)] in this age group having a 100‐ to 200‐fold increased incidence as compared to the general population.^4^ Nevertheless, as such disorders are rare, randomized trials from which to derive optimal treatment are lacking, although prognosis has improved by employing a step‐wise process of reduction in immunosuppression, immune therapy with rituximab, mild chemotherapy, and, more recently, EBV‐specific cytotoxic T‐lymphocytes.^5^

EBV infection is identified in most cases of PTLD and plays an important role in the etiology and pathophysiology of this disorder.^6^, ^7^ The highest risk for PTLD after SOT results from transplantation of an EBV‐positive organ donor to an EBV‐naïve recipient.^8^, ^9^, ^10^, ^11^, ^12^ For PTLD following HSCT, a converse serological EBV constellation of donor and recipient is an established risk factor.^13^ Rapidly rising and/or newly appearing EBV load after SOT and HSCT is utilized by most physicians to reduce immunosuppression and/or begin pre‐emptive therapy with rituximab, respectively. Although rare, fulminant PTLD is more commonly seen following HSCT.^5^

The ability of EBV to transform normal B‐lymphocytes into continuously growing lymphoblastoid cells is attributed to its latent proteins. Among them, LMP1 and EBNA2 have been extensively studied.^14^, ^15^ In particular, it is well established that EBNA2 is crucial for the viral transformation of B‐lymphocytes.^16^ Although there are observations indicating that the presence of EBV is correlated with higher expression of PD‐L1 in DLBCL and BL, there are, to our knowledge, no comprehensive reports on its expression in pediatric and adolescent patients with PTLD, particularly, not on the entire spectrum of PTLD categories, including early, non‐destructive lesions.

Post‐transplantation immunosuppression in an EBV‐positive patient reduces the activity of the patient's EBV‐specific cytotoxic T‐cell surveillance, which increases the chances of uncontrolled proliferation of EBV‐infected B‐cells and subsequent progression to PTLD.^17^, ^18^ Management of such cases can be challenging and must balance the goal of PTLD eradication against the risks of graft rejection, graft‐versus‐host disease, further delays in immune reconstitution and life‐threatening infections, amongst others.^19^

Immune checkpoints regulate T‐cell responses to maintain self‐tolerance. They deliver co‐stimulatory and co‐inhibitory signals to T‐lymphocytes.^20^ PD‐L1 (programed death‐ligand 1), mainly expressed by antigen‐presenting cells, engages its receptor PD1 on T‐cells, to provide a growth inhibitory signal, thereby evading elimination by T‐cells. Different tumors express high PD‐L1 to evade immune recognition and, consequently, inhibition of PD‐L1 and PD1 have become important targets of cancer immunotherapy.^20^

In this study, we investigated the expression of PD‐L1 and PD1 in the different categories of PTLD in 21 pediatric SOT and HSCT patients, allowing not only an inter‐individual comparison of the results, but also the analysis of the course of expression on an intra‐individual level in patients with consecutively collected matched tumor samples. Based on our results, we propose checkpoint inhibition as a potential treatment option for children and adolescents with PTLD if standard immune‐ and chemotherapy is not successful or impossible.

## 2 PATIENTS AND METHODS

### 2.1 Patients

Twenty‐seven consecutive cases of pediatric and adolescent PTLD were retrieved from the patient's databases of the St. Anna Children's Hospital, the Department of Pediatrics and Adolescent Medicine, and the Department of Pathology of the Medical University of Vienna, Austria. Twenty‐one/27 (78%) cases (SOT, n = 17; HSCT, n = 4, Table 1) with available formalin‐fixed and paraffin‐embedded (FFPE) tissue blocks were included in this study. Median age at transplant was 6.59 years (range: 0.64‐18.56 years). It was 11.69 years (range: 1.86‐21.81 years) at PTLD diagnosis and the male‐to‐female ratio was 12:9. The study was conducted in accordance with the declaration of Helsinki after the approval by the ethics committee and institutional review boards.

**Table 1:** Main characteristics of the 21 patients with post‐transplantation lymphoproliferative disease

Pt. No.	Age (years)	Gender	Type of TX	Diagnosis	Subtype of PTLD	Course of disease	Localisation	EBV status	PD‐L1 expression				PD1 expression		
									Tumor cells	Macrophages	Intensity	Percentage^d^	Tumor cells	Lymphocytes	Percentage^d^
1	12.18	Female	HSCT	m‐PTLD	DLBCL^a^	Primary disease	CNS	+	+	‐	3	40%	‐	‐	/
2	12.11	Male	HSCT	m‐PTLD	DLBCL^a^	Primary disease	Skin	+	‐	+	2	20%	‐	+	5%
3	7.57	Female	HSCT	m‐PTLD	DLBCL^a^	Primary disease	LN cervical	+	‐	+	3	40%	‐	+	30%
4	11.69	Male	Heart	m‐PTLD	DLBCL^a^	Primary disease	Nasal sinus	+	‐	+	2	20%	‐	+	5%
				m‐PTLD	DLBCL^a^	Relapse	Epipharynx	+	‐	+	1	10%	‐	‐	/
5	21.88	Male	Heart	m‐PTLD	DLBCL^b^	Primary disease	LN retroperitoneal	‐	‐	+	1	5%	‐	+	10%
6	16.28	Male	Kidney	m‐PTLD	DLBCL^a^	Primary disease	Colon	‐	+	‐	3	80%	‐	‐	/
7	6.19	Male	Kidney	m‐PTLD	DLBCL^a^	Primary disease	LN cervical	+	+	‐	3	70%	‐	‐	/
8	19.19	Female	Lung	m‐PTLD	DLBCL^a^	Primary disease	Epipharynx	+	+	‐	3	30%	‐	‐	/
9	17.27	Female	Kidney	m‐PTLD	DLBCL^a^	Primary disease	LN cervical	+	+	+	2	40%	‐	‐	/
10	17.46	Male	Kidney	m‐PTLD	DLBCL^a^	Primary disease	Palatine	+	+	‐	3	70%	‐	+	5%
11	16.63	Male	Liver	Non‐destructive PTLD	PH	Primary disease	Colon	+	‐	+	1	10%	‐	‐	5%
				m‐PTLD	PBL	Progression	Ileum	+	n.a.	n.a.	n.a.	n.a.	n.a.	n.a.	n.a.
12	21.81	Male	Kidney	m‐PTLD	PBL^c^	Primary disease	Colon	‐	‐	‐	/	/	‐	‐	/
13	7.12	Female	Liver	m‐PTLD	BL ^c^	Primary disease	Ileum	+	‐	+	1	10%	‐	+	15%
14	8.78	Female	Liver	m‐PTLD	MALT l.	Primary disease	Lung	+	‐	‐	/	/	‐	‐	/
15	13.04	Female	Heart	p‐PTLD		Primary disease	LN cervical	+	‐	+	2	20%	‐	+	20%
				p‐PTLD		Persistence	Colon	+	+	+	3	30%	‐	+	15%
				m‐PTLD	DLBCL	Progression	Esophagus	+	n.a.	n.a.	n.a.	n.a.	n.a.	n.a.	n.a.
16	8.37	Male	HSCT	p‐PTLD		Primary disease	Stomach	+	‐	+	2	20%	‐	+	20%
17	2.52	Male	Kidney	p‐PTLD		Primary disease	Colon, stomach	+	‐	+	2	20%	‐	+	15%
				p‐PTLD		Persistence	Rectum	+	+	+	2	20%	‐	+	15%
				Non‐destructive PTLD	FFH	Regression	Duodenum	+	‐	+	1	5%	‐	+	20%
18	8.85	Female	Kidney	p‐PTLD		Primary disease	LN cervical	+	n.a.	n.a.	n.a.	n.a.	n.a.	n.a.	n.a.
				p‐PTLD		Persistence	Colon, stomach	+	‐	‐	/	/	‐	+	10%
				Non‐destructive PTLD	FFH	Regression	Tonsil	+	‐	‐	/	/	‐	+	20%
19	3.60	Male	Kidney	Non‐destructive PTLD	IM	Primary disease	Tonsil	+	‐	+	2	20%	‐	+	20%
				p‐PTLD		Progression	LN cervical	+	+	+	3	30%	‐	+	10%
20	1.86	Male	Kidney	Non‐destructive PTLD	IM	Primary disease	LN cervical	+	‐	+	1	15%	‐	+	15%
21	2.92	Female	Liver	Non‐destructive PTLD	FFH/PH	Primary disease	Duodenum	+	‐	+	1	5%/‐	‐	+	20%/5%
				Non‐destructive PTLD	FFH/PH	Persistence	Duodenum, colon	+	‐	+	1	5%/‐	‐	+	20%/10%
				Non‐destructive PTLD	FFH/PH	Persistence	Colon	+	‐	+	2	5%/15%	‐	+	20%/ 10%

Abbreviations: Pt. No., patient number; PTLD, post‐transplantation lymphoproliferative disease; TX, transplantation; HSCT, hematopoietic stem cell transplantation; m‐PTLD, monomorphic PTLD; p‐PTLD, polymorphic PTLD; DLBCL, diffuse large B‐cell lymphoma; BL, Burkitt's lymphoma; PH, plasma cell hyperplasia; PBL, plasmablastic lymphoma; MALT l., mucosa‐associated lymphoid tissue lymphoma; FFH, florid follicular hyperplasia; IM, infectious mononucleosis; CNS, central nervous system; LN, lymph node; EBV, Epstein‐Barr virus; n.a., not available; +, positive; ‐, negative.

a Non‐germinal center B‐cell subtype, *c‐MYC* translocation‐negative.

b Germinal center B‐cell subtype, *c‐MYC* translocation‐negative.

c *c‐MYC* translocation‐positive.

d Proportion of immunolabeled cells in relation to total cellularity.

### 2.2 Histopathological analyses

All hematoxylin and eosin stained slides of each patient were reviewed and diagnoses were established according to the 2016 revised WHO (World Health Organization) Classification of Hematopoietic and Lymphoid Tissues by two authoring hematopathologists (AIS, ISK).^6^ Representative blocks of whole tissue sections (n = 14) and biopsies (n = 18) were chosen for further immunohistochemical analyses. In three of 32 cases (Pt. #17, #18, #21), there were two samples (gastro‐intestinal tract) from the same time point available, displaying identical results each, so that they were not counted separately for further analysis (final number of samples: 29; Figure 1). Immunophenotypic classification of DLBCL into germinal center B‐cell (GCB) vs non‐GCB subtype was done by immunohistochemistry according to the Hans algorithm.^21^ *C‐MYC* status of monomorphic PTLD was assessed by interphase fluorescent in‐situ hybridization on 4 µm slides of FFPE tissue using the LSI *c‐MYC* Dual Color Break Apart Rearrangement Probe (Vysis, Abbott Laboratories, Illinois, USA) according to the manufacturer's instructions. EBV status was assessed by in situ hybridization (Bond EBER Probe PB0589, Leica Biosystems, Nussloch, Germany) using the automated Leica Bond III Immunostainer (Leica Biosystems).

**Figure 1:**
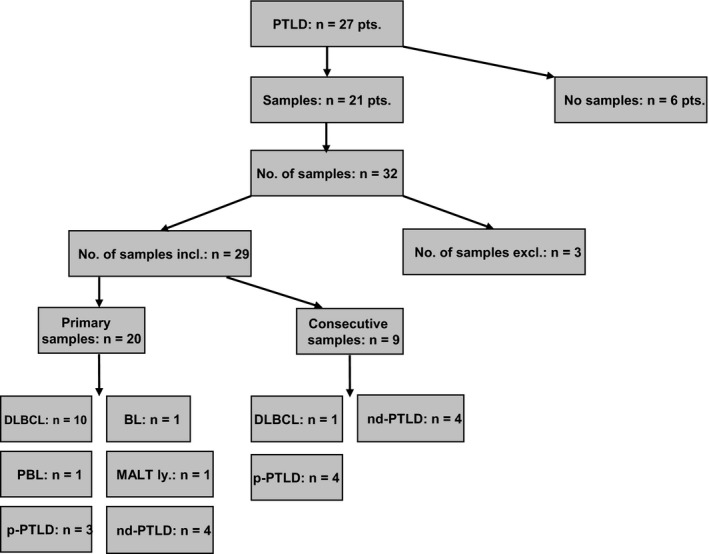
Flow chart of the 21 patients with PTLD and available tumor samples (n = 29). PTLD, post‐transplantation lymphoproliferative disease; No., number; incl., included; excl., excluded; DLBCL, diffuse large B‐cell lymphoma; BL, Burkitt's lymphoma; PBL, plasmablastic lymphoma; MALT ly., mucosa‐associated lymphoid tissue lymphoma; p‐PTLD, polymorphic PTLD; nd‐PTLD, non‐destructive PTLD

### 2.3 Immunohistochemical analyses

Immunohistochemical analyses were performed on 2 µm slides of FFPE tissue using the automated Leica Bond III Immunostainer. The following primary antibodies were used: for PD‐L1: DAKO, monoclonal mouse anti‐human antibody, clone 22C3, Glostrup, Denmark, and for PD1 detection: CellMarque, monoclonal mouse anti‐human antibody, clone NAT 105, Darmstadt, Germany. Heat induced epitope retrieval was done with Bond Epitope Retrieval Solution (Solution 2 for PD‐L1 and Solution 1 for PD1) for 20 minutes. Primary antibodies were incubated for 30 minutes. Staining was done using the Bond Polymere Refine Detection Kit DS9800 according to the manufacturer's guidelines. Tissue sections were counterstained with hematoxylin.

Double immunohistochemical staining for PAX5 (DAKO, monoclonal mouse anti‐human antibody, clone M7307) und PD‐L1 were done in selected cases. Stains were performed in a sequential manner using the Leica Bond III Immunostainer platform with the protocol described above except for counterstaining, which was performed only once at the end. PAX5 antibody was used as the first agent and developed with brown color followed by a red color staining for PD‐L1 using the BOND Polymer Refine Red Detection Kit DS9390. Immunohistochemistry was assessed by two hematopathologists together on a multi‐headed microscope. Several high‐power fields (at least 10 at whole tissue sections, at least three at biopsies) were examined and hot spots were defined. A distinct membranous staining on cells with medium or high intensity was rated positive. Lymphocytes in germinal centers served as internal positive controls for PD1. The amount of immunolabeled cells with respect to total cellularity (for PD1 and PD‐L1) and of the stained tumor cells (for PD‐L1) was estimated in each case by the two hematopathologists. Analyses were done on hot spots. The cut off level for PD1 positivity was set at ≥ 20% immunolabeled cells. According to previous publications, different cut‐off levels for PD‐L1 positivity were applied.^22^, ^23^, ^24^ Accordingly, tumor cells were rated positive if > 5% of tumor cells exhibited specific membranous staining. The tumor microenvironment was defined as PD‐L1‐positive if at least 20% of the total tissue cellularity (malignant and non‐malignant cells) showed moderate to strong PD‐L1 staining. The discrimination of tumor cells vs non‐malignant cells was primarily done on morphological aspects; in cases of uncertainty, the B‐cell nature and the amount of PD‐L1 staining was confirmed by PAX5/PD‐L1 double staining. According to previous publications, we defined the density of PD‐L1‐positive cells semi‐quantitatively: scattered PD‐L1‐positive cells were rated as low density, loose but widespread infiltration was considered moderate density and widespread distention was defined as high density.^23^

## 3 RESULTS

### 3.1 Patient's characteristics of the 21 PTLD cases

Patient's characteristics are presented in Table 1 and Supporting Information Table S1. Basic parameters of therapy, response and outcome are shown in Table 2. Six/21 (29%) patients had sequential biopsies during the course of disease available including one with no material from primary disease for further immunostaining. As for primary disease, 8 of 21 (33%) had non‐destructive or polymorphic PTLD (p‐PTLD) and 13 of 21 (67%) patients had monomorphic PTLD (m‐PTLD). Among the latter, DLBCL comprised the majority with 10 cases being identified (Pt. #1‐10). One patient was diagnosed as plasmablastic lymphoma (PBL) (Pt. #12), 1 as BL (Pt. #13) and 1 as EBV‐positive mucosa‐associated lymphoid tissue (MALT) lymphoma (Pt. #14). One patient with DLBCL (Pt. #15) and 1 patient with PBL (Pt. #11) suffered from their m‐PTLD in terms of progression of disease, 54 and 4 months after the diagnosis of a polymorphic and non‐destructive PTLD, respectively. Unfortunately, both samples of m‐PTLD were only very small biopsies with insufficient material for further immunohistochemical analyses.

**Table 2:** Treatment, response and outcome of the 21 patients with post‐transplantation lymphoproliferative disease

Pt. No.	Type of TX	Diagnosis	Subtype of PTLD	Reduction of immunosuppression	Rituximab	Chemotherapy	Response	Death	Cause of death	Follow‐up^c^	
1	HSCT	m‐PTLD	DLBCL	Yes	Yes	No	FOP	Yes	HSCT‐related	1.19 y	
2	HSCT	m‐PTLD	DLBCL	Yes	Yes	Yes	PD	Yes	PTLD‐therapy related		0.10 y
3	HSCT	m‐PTLD	DLBCL	Yes	Yes	No	FOP	Yes	HSCT‐related	0.30 y	
4	Heart	m‐PTLD	DLBCL	Yes	Yes	No	FOP	No	/	/	
		m‐PTLD	DLBCL	n.a.	Yes	n.a.	n.a.	n.a.	n.a.	LFU	
5	Heart	m‐PTLD	DLBCL	No	Yes	Yes	FOP	No	/	4.10 y	
6	Kidney	m‐PTLD	DLBCL	No	Yes	Yes	FOP	No	/	9.83 y	
7	Kidney	m‐PTLD	DLBCL	Yes	Yes	No	FOP	No	/	LFU	
8	Lung	m‐PTLD	DLBCL	Yes	Yes	Yes	FOP	No	/	0.96 y	
9	Kidney	m‐PTLD	DLBCL	Yes	Yes	No	FOP	No	/	6.46 y	
10	Kidney	m‐PTLD	DLBCL	Yes	Yes	Yes	FOP	No	/	10.87 y	
11	Liver	Non‐destructive PTLD	PH	Yes	Yes	No	PD	No	/	1.16 y	
		m‐PTLD	PBL	Yes	No	Yes	FOP	No	/		
12	Kidney	m‐PTLD	PBL	Yes	No^a^	Yes	PD	Yes	PTLD‐related	0.15 y	
13	Liver	m‐PTLD	BL	Yes (incl. total resection)	No	No	FOP	No	/	19.56 y	
14	Liver	m‐PTLD	MALT lymphoma	Yes	No	Yes	FOP	No	/	2.75 y	
15	Heart	p‐PTLD		Yes	Yes	Yes	FOP	No	/	/	
		p‐PTLD^b^		Yes	Yes	Yes	PR	Yes	PTLD‐related	4.53 y	
		m‐PTLD^b^	DLBCL	Yes	Yes	Yes	PR	Yes	PTLD‐related		
16	HSCT	p‐PTLD		No	Yes	No	FOP	No	/	1.02 y	
17	Kidney	p‐PTLD		Yes	No	No	n.a.	No	/	1.45 y	
		p‐PTLD		Yes	No	No	n.a.	No	/		
		Non‐destructive PTLD	FFH	No	No	No	n.a.	No	/		
18	Kidney	p‐PTLD		Yes	Yes	No	FOP	No	/	7.81 y	
		p‐PTLD		n.a.	n.a.	n.a.	n.a.	n.a.	/		
		Non‐destructive PTLD	FFH	No (only total resection)	No	No	FOP	No	/		
19	Kidney	Non‐destructive PTLD	IM	n.a.	n.a.	n.a.	n.a.	No	/	0.63 y	
		p‐PTLD		Yes	Yes	Yes	PD	Yes	PTLD‐related		
20	Kidney	Non‐destructive PTLD	IM	Yes	No	No	FOP	No	/	2.54 y	
21	Liver	Non‐destructive PTLD	FFH/ PH	Yes	No	No	SD	No	/	1.38 y	
		Non‐destructive PTLD	FFH/ PH	Yes	No	No	SD	No	/		
		Non‐destructive PTLD	FFH/ PH	Yes	Yes	No	n.a.	No	/		

Abbreviations: Pt. No., patient number; PTLD, post‐transplantation lymphoproliferative disease; TX, transplantation; HSCT, hematopoietic stem cell transplantation; m‐PTLD, monomorphic PTLD; p‐PTLD, polymorphic PTLD; DLBCL, diffuse large B‐cell lymphoma; BL, Burkitt's lymphoma; PH, plasma cell hyperplasia; PBL, plasmablastic lymphoma; MALT, mucosa‐associated lymphoid tissue; FFH, florid follicular hyperplasia; IM, infectious mononucleosis; FOP, freedom of progression, PD, progressive disease; PR, partial response; SD, stable disease; n.a., not available; y, years; LFU, lost to follow‐up.

a pt. received Brentuximab‐vedotin.

b Both samples were from the same time point of relapse diagnosis.

c Follow‐up was calculated from date of diagnosis of primary disease until date of death for succumbed patients and until date of last follow‐up for surviving patients, respectively. Two patients were lost to follow‐up.

Among the eight patients with non‐m‐PTLD, four patients (Pt. #15‐18) were primarily diagnosed as p‐PTLD. Two showed regression of their PTLD category at subsequent biopsies with the morphology of a non‐destructive florid follicular hyperplasia (FFH) PTLD (Pt. #17‐18), one patient progressed to DLBCL (Pt. #15) and patient #16 did not have further sampling. Of note, one patient developed p‐PTLD as a progressive disease in a cervical lymph node 8 months after the diagnosis of non‐destructive infectious mononucleosis (IM) PTLD in the tonsil (Pt. #19).

Among the eight patients with non‐m‐PTLD, another four patients (Pt. #11, #19‐21) were primarily diagnosed as non‐destructive PTLD, including 1 plasma cell hyperplasia (PH) PTLD prior to the diagnosis of PBL (Pt. #11), 1 IM PTLD prior to the diagnosis of p‐PTLD (Pt. #19), 1 without further sampling (IM PTLD, Pt. #20) and 1 case (Pt. #21) which showed a persisting PTLD category at two subsequent biopsies 3 and 7 months later, with all specimens showing the morphology of both FFH and PH. Of note, two patients showed non‐destructive PTLD (FFH) at later time points of the disease course (three and 31 months, respectively) after having had p‐PTLD as the primary disorder (Pt. #17‐18).

**Figure 2:**
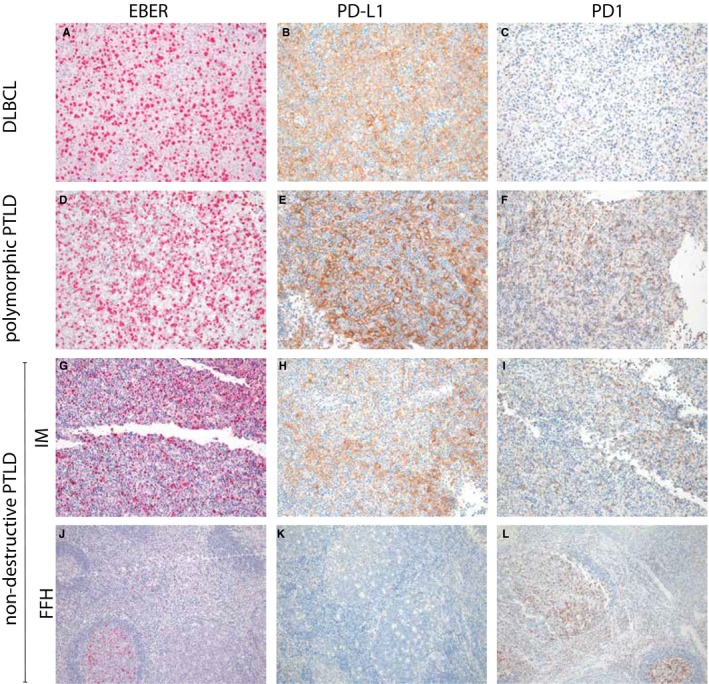
EBER, PD‐L1, and PD1 staining of m‐PTLD (DLBCL), p‐PTLD and non‐destructive PTLD (IM and FFH). DLBCL (Pt. #7) displaying strong EBER (A, 200×) and PD‐L1 (B, 200×) immunoreactivity, with only few intermingled PD1‐positive lymphocytes (C, 200×); p‐PTLD (Pt. #15) with EBV positivity (D, 200×), PD‐L1‐positive microenvironment (E, 200×) and moderate PD1‐positive lymphocytes (F, 200×); IM PTLD (Pt. #19) with EBV positivity (G, 200×), moderate PD‐L1‐positive macrophages (H, 200×) and moderate PD1‐positive lymphocytes (I, 200×); FFH PTLD (Pt. #18) with few EBV‐positive cells (J, 100×), few PD‐L1‐positive macrophages (K, 100×) and moderate PD1‐positive lymphocytes in the intra‐ and also peri‐follicular region (L, 100×)

All samples analyzed in the present study except for two cases of DLBCL (Pt. #5‐6) and one patient with PBL (Pt. #12) were EBV‐positive (Figure 2A,D,G,J). Immunohistochemically, cell of origin could be determined in all 11 DLBCL samples, with 10 showing a non‐GCB subtype and 1 EBV‐negative sample exhibiting a GCB phenotype. All but the 2 patients with PBL (Pt. #11, #12) and 1 with p‐PTLD (Pt. #16) were CD20‐positive (Table S1). A *c*‐*MYC* translocation was detected in 1 patient each with BL (Pt. #13) and PBL (Pt. #12). Ten of 11 DLBCL samples investigated were negative for a *c‐MYC* translocation (Table S1). The remaining sample was from a DLBCL relapse initially negative for a *c‐MYC* rearrangement (Pt. #4).

### 3.2 PD‐L1 expression in 20 primary and 9 consecutive tumor samples

We investigated PD‐L1 expression in all 20 primary samples and in consecutive specimens of 6 patients (3 with 2 and 3 with 1 consecutive specimens). Thus, overall, we examined 29 specimens: DLBCL, n = 11; BL, n = 1; PBL, n = 1; MALT lymphoma, n = 1; p‐PTLD, n = 7; non‐destructive early lesions, n = 8.

**Figure 3:**
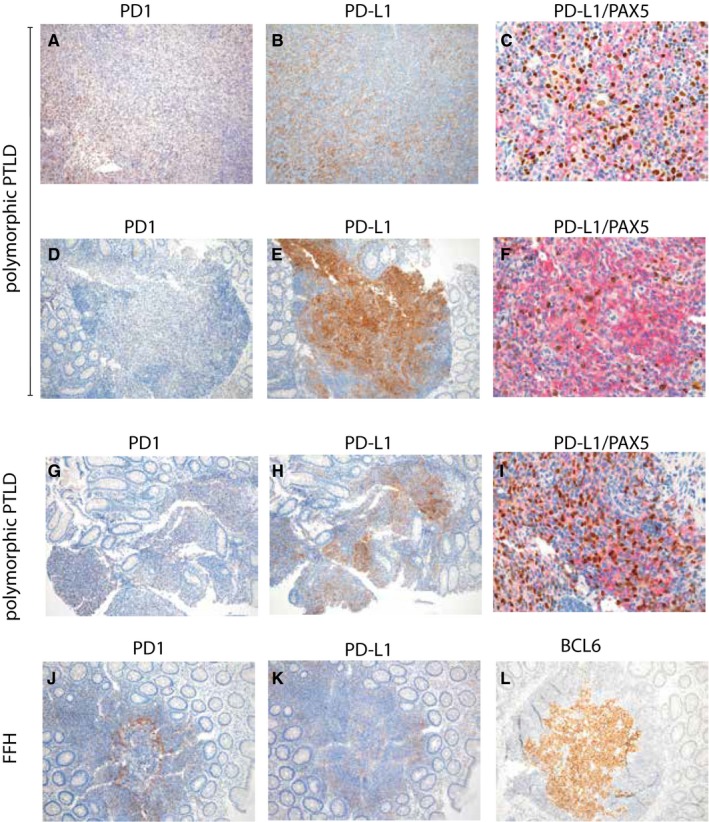
PD1, PD‐L1, and PD‐L1/PAX5 staining of two patients with p‐PTLD (one with persisting p‐PTLD and one with regression to FFH). Patient #15 (p‐PTLD): A–C represent sections from the first lymph node specimen, D–F form the subsequent colon biopsy. The patient showed an increase in PD‐L1‐positive macrophages in the course of disease (B, 100×, E, 100×) and a novel PD‐L1 staining of B‐cell blasts with co‐labeling of nuclear PAX5 (brown) and membranous PD‐L1 (red, F, 400×) which was not detectable in the prior specimen (C, 400×, no PAX5/PD‐L1 co‐labeling). The number of PD1‐positive lymphocytes decreased (A, 100 × D, 100×). Patient #17 (p‐PTLD): The number of PD‐L1‐positive cells decreased (H, 100×, I, 400×, co‐staining of PAX5‐positive B‐blasts with PD‐L1, K, 100×), while that of PD1‐positive cells increased (G, 100×, FFH: J, 100×, BCL6‐staining of the germinal center region, L, 100×) with regression to FFH

The results of PD‐L1 immunohistochemical analyses are provided in Table 1. PD‐L1 expression was found on both tumor cells and macrophages. Tumor cells showed PD‐L1 positivity in 6 of 11 DLBCL samples (Figure 2B) and in 3 of 7 p‐PTLD samples (Figure 3E,F,H,I). Due to variable cellular composition and challenging morphological assessment of the polymorphic infiltrates, PD‐L1 positivity of tumor cells was confirmed by double PAX5/PD‐L1 staining in the latter (Figure 3F,I). None of the non‐destructive early lesions or non‐DLBCL m‐PTLD samples expressed PD‐L1 on tumor cells.

A PD‐L1‐positive microenvironment was detected in 4 of 11 DLBCL samples (one with additional PD‐L1 tumor cell expression, pt. #9), 6 of 7 p‐PTLD samples (Figures 2E and 3B; three with additional PD‐L1 tumor cell expression, pt. #15, 17, 19, Figure 3E,F,H,I) and in 1 of 8 specimens of non‐destructive PTLD (Pt. #19, Figure 2H). None of the other 7 non‐destructive early lesions or non‐DLBCL m‐PTLD samples expressed PD‐L1 in the microenvironment.

**Figure 4:**
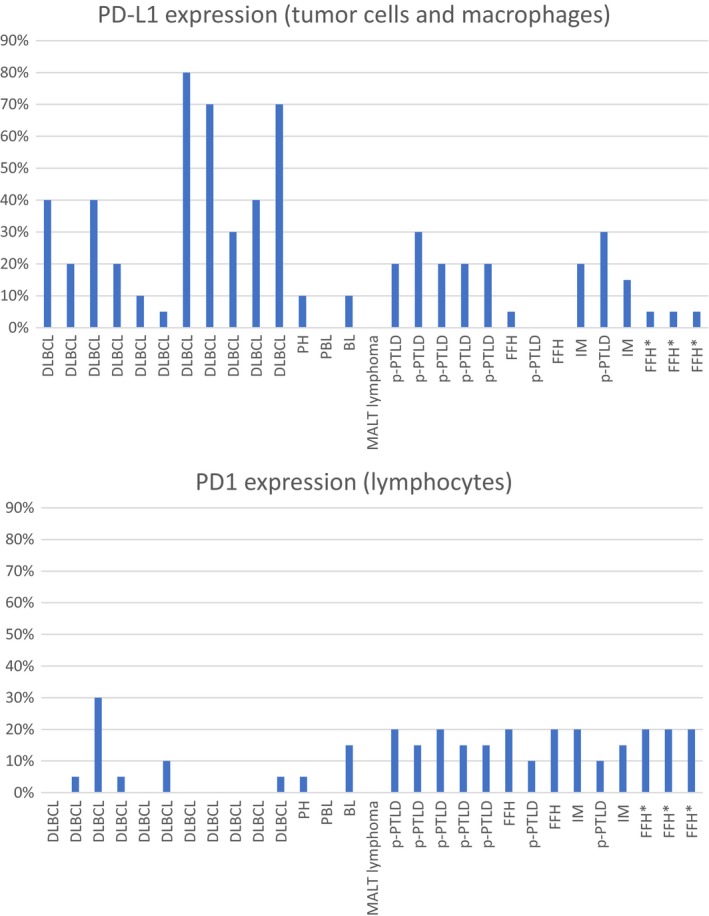
Bar diagram of PD‐L1 vs PD1 expression of the 29 PTLD samples. DLBCL, diffuse large B‐cell lymphoma; PH, plasma cell hyperplasia; PBL, plasmablastic lymphoma; BL, Burkitt's lymphoma; MALT, mucosa‐associated lymphoid tissue; p‐PTLD, polymorphic post‐transplantation lymphoproliferative disorder; FFH, florid follicular hyperplasia; IM, infectious mononucleosis. *All three samples also contained PH, however, their expression of PD‐L1 and PD1 is not depicted in the diagrams, but only for the FFH

In summary, PD‐L1 expression regardless of cell type was observed in 9 of 11 DLBCL samples (Figures 2B and 4), the percentage of positive cells ranged from 20%‐80%, with higher levels in samples with tumor cell positivity (Table 1). None of the remaining 3 monomorphic non‐DLBCL PTLD exhibited PD‐L1 positivity, neither on the tumor cells, nor in the microenvironment, in an appropriate concentration (Figure 4).

PD‐L1 expression regardless of cell type was found in 6 of 7 p‐PTLD specimens with a percentage of positive cells ranging from 20%‐30% (Figures 2E, 3B,E,H and 4). In all 6 samples macrophages were PD‐L1‐positive; however, in three cases PD‐L1 positivity was also detected on B‐cell blasts (Figure 3F,I). Two p‐PTLD cases comprised few intermingled Hodgkin and Reed‐Sternberg‐like cells which displayed PD‐L1 positivity in one case.

With a single exception (1/8; pt. #19), non‐destructive lesions did not reveal PD‐L1 positivity (Figure 4). In 6 of the other 7 samples PD‐L1‐positive macrophages could be detected only sparsely, but with a distinct distribution: in FFH lesions they were accentuated within and around germinal centers (Figures 2K and 3K), in IM lesions they clustered with EBV‐positive B‐cell blasts (Figure 2G,H), and in PH samples they were regularly distributed. The highest levels of PD‐L1 expression was detected in IM (15%), the lowest level in FFH (5%), and in PH lesions the positivity ranged from 10%‐15%.

### 3.3 PD1 expression in 20 primary and 9 consecutive tumor samples

In the 29 samples of PTLD analyzed, PD1 expression was only detected on lymphocytes but not on tumor cells (Table 1 and Figure 4). The density of PD1‐positive cells varied among PTLD subtypes and was higher in non‐destructive PTLD (6 of 8 samples with a positivity of 20%), in particular, in FFH lesions, where they clustered within and also around germinal centers (Figures 2L and 3J). Polymorphic PTLD showed regularly interspersed PD1‐positive lymphocytes at a moderate density with a positivity of 10%‐15% in 5/7 samples (Figures 2F and 3D,G); 2 of 7 with 20% positivity (Figure 3A). Among the m‐PTLD samples 8 of 14 showed no expression of PD1 (Figure 2C), 5 of 14 a positivity between 5%‐15%, and only 1 patient was truly positive with 30% positivity.

### 3.4 PD‐L1 and PD1 expression in consecutive tumor samples of 6 patients

In individual patients, the density of PD‐L1 expression changed in the course of disease and according to the PTLD category (Table 1): the amount of PD‐L1 expression increased with persistence of the PTLD category [Pt. #15 (p‐PTLD) by 10% and pt. #21 (PH) by 15%] and with progression of the PTLD category (Pt. #19: non‐destructive to p‐PTLD by 10%). The PD‐L1 expression decreased with regression of the PTLD category (Pt. #17: polymorphic to non‐destructive PTLD by 15%.) In patient #18 PD‐L1 expression remained negative with regression of the PTLD category from p‐PTLD to non‐destructive PTLD. In remaining patient #4 relapse of DLBCL showed reduction of PD‐L1 expression on the macrophages by 10%, while the tumor cells remained PD‐L1‐negative.

B‐cell tumor cells turned from originally PD‐L1‐negative to positive in the course of persisting disease in two patients with p‐PTLD [Pt. #15 (Figure 3C,F) and #17 (Figure 3I)], and after progression from non‐destructive to p‐PTLD in 1 patient (Pt. #19), respectively. For example, patient #15 developed primary p‐PTLD in a cervical lymph node 150 months after heart transplantation (Figure 3A‐C). A colon biopsy taken 54 months later (Figure 3D‐F) demonstrated persistence of the PTLD category, but an increase of the number of PD‐L1‐positive macrophages from initially 20% to 30% (Figure 3B,E) and, in addition, a novel PD‐L1 positivity of the B‐cell blasts (Figure 3F); PD1 expression decreased (20% to 15%, Figure 3A,D). This patient progressed to DLBCL at the same time in the esophagus; however, unfortunately, the biopsy specimen was too small for further PD‐L1 testing.

In addition, we noticed a decrease in PD‐L1 expression with regression of the PTLD category. For example, patient #17 initially presented with p‐PTLD of the colon and stomach after kidney transplantation with 20% PD‐L1 positivity in each specimen. A duodenal biopsy 3 months later revealed FFH PTLD with just 5% PD‐L1‐positive macrophages (Figure 3K), and, of note, PD‐L1 negativity of the B‐cell blasts, which were PD‐L1‐positive in a sample of the rectum (p‐PTLD) (Figure 3H,I) taken simultaneously to the duodenal biopsy.

Compared to the dynamics of PD‐L1 expression, PD1 expression also changed according to the PTLD subtype, that is, the amount of PD1‐positive cells increased with regression (Pt. #17 and #18: p‐PTLD to non‐destructive PTLD by 5% and 10%, respectively; Figure 3G,J) and decreased with progression of the PTLD category (Pt. #19: non‐destructive PTLD to p‐PTLD by 10%).

## 4 DISCUSSION

Post‐transplantation lymphoproliferative disease represents a heterogeneous spectrum of diseases ranging from polyclonal lymphoid proliferations to monoclonal mature B‐cell lymphoma such as DLBCL and BL. Previous PD‐L1/PD1 studies mainly focused on monomorphic and, with few exceptions, also on PTLD in adults. Herein, we provided the first comprehensive pediatric study cohort assessing systematically PD‐L1 and PD1 expression in all WHO categories of PTLD such as non‐destructive, polymorphic and monomorphic PTLD. Using PD1 as an additional marker we also focused on tumor vs microenvironment interactions, and additionally, also evaluated consecutively collected matched tumor samples of several individual patients to reproduce inter‐individual observations on an intra‐individual basis.

We clearly found that the degree of PD‐L1 expression inversely correlated with that of PD1 expression in surrounding tissues of all PTLD categories, with the highest PD‐L1 expression in DLBCL [9 of 11 (82%) vs PD1 expression in 1 of 11 (9%) cases] and in p‐PTLD [6 of 7 (86%) vs PD1 expression in 2 of 7 (29%) cases], and the lowest expression in non‐destructive lesions [1 of 8 (12.5%) vs PD1 expression in 6 of 8 (75%) cases]. We also clearly demonstrated for 6 patients with matched tumor samples available not only a maintained inverse correlation of PD‐L1 and PD1 expression, but also very well‐correlating dynamics of PD‐L1 and PD1 expression with progression, persistence, and regression of the WHO PTLD category.

Depending on the cut‐off levels used for positivity, previous studies on adult DLBCL indicated a wide range of 26%‐75% of patients to be PD‐L1‐positive.^25^, ^26^, ^27^, ^28^ In studies including immunocompromised patients, similar to our patient cohort, the proportion of PD‐L1‐positive B‐cell lymphoma cases was rather comparable to that seen by us.^22^ Similar to our observations with inter‐ and intra‐individual changes of PD‐L1 and PD1 expression according to the PTLD category, Carreras et al^29^ found higher levels of PD1‐positive cells in low‐grade and lower levels in high‐grade follicular lymphoma (FL) and, intriguingly, an intra‐individual decrease of PD1‐positive cells when FL transformed to DLBCL. Of note, Dilly‐Feldis et al^30^ reported a transition from initially negative to positive PD1 labeling of intra‐tumoral lymphocytes in five of nine relapsed/refractory cases of pediatric classical Hodgkin's lymphoma (cHL) and a trend toward a more intense PD‐L1 staining of tumor cells at relapse.

PD1‐positive lymphocytes were, with one exception, only sparsely detected in our 11 cases of DLBCL. Kiyasu et al^31^ investigated PD1 expression in 236 patients with DLBCL and found lower numbers of PD1‐positive cells in DLBCL of the activated B‐cell subtype [10 of 11 (91%) of our cases: non‐GCB subtype] as well as in DLBCL with high (≥30%) PD‐L1 tumor cell positivity, which reflects our findings of an inverse behavior of the two proteins.^31^ However, conflicting results with a positive correlation of PD1‐positive cells with PD‐L1 expression of tumor cells and/or macrophages have been reported by others, as well.^25^, ^32^

As for p‐PTLD, PD‐L1 positivity in 6 of 7 cases within a range of 20%‐30% was below the level detected in our DLBCL samples (20%‐80%). Intermingled Hodgkin‐ and Reed‐Sternberg‐like cells, which were present in two p‐PTLD samples, displayed PD‐L1 positivity in one of them. A strong PD‐L1 expression in the majority of the malignant cells is characteristic and usually observed in 70%‐87% of cHL samples.^33^ Although some overlapping features to cHL were present in our p‐PTLD cases, stringent criteria allowing the diagnosis of cHL were not met and, thus, they were finally classified as p‐PTLD. PD1‐positive lymphocytes were regularly distributed in a moderate density in the p‐PTLD samples. Of note, there was no case with an amount below 10% PD1‐positive lymphocytes.

To the best of our knowledge, there are no studies on pediatric patients and only a single report dealing with PD‐L1 und PD1 expression in p‐PTLD. Kinch et al investigated the expression patterns of PD1, PD‐L1, and PD‐L2 in 81 PTLD patients after SOT and found positivity for PD‐L1 in two and PD1 positivity in three of five p‐PTLD samples, respectively.^24^ In our study, we applied the same cut off levels for PD‐L1 expression and observed PD‐L1 positivity in 6 of 7 patients and PD1 positivity in all samples (although there is limited evidence for definite cut‐off levels for PD1 expression). Reasons explaining this discrepancy are lacking, although, reagent variability could perhaps account for this difference.

With only one sample with a PD‐L1‐positive microenvironment, non‐destructive PTLD exhibited the lowest rate of PD‐L1 expression, but the highest PD1 expression. The PD‐L1‐positive microenvironment was observed in 1 case of IM PTLD where a clustering of PD‐L1‐positive macrophages with EBV‐positive cells was evident, pointing out the pathogenetic contribution of EBV (Figure 2G,H). Florid follicular hyperplasia PTLD exhibited the highest rate of PD1‐positive cells with a peculiar distribution (Figures 2L and 3J). As PD1 expression is mainly found in follicular germinal center T‐cells in lymphoid tissues, the intra‐follicular PD1 expression is not surprising and probably represents a physiological situation. But aside from that, we also noticed a distinct peri‐follicular pattern. A main intra‐follicular expression of PD1 has been reported in various studies of FL where only sparsely stained cells were found in the extra‐follicular compartment.^29^, ^34^ However, Smeltzer et al^35^ also observed peri‐follicular PD1‐positive T‐cells in a diffuse distribution in one third of patients with FL that transformed into DLBCL later on. Of note, the diffuse distribution rather than the quantity of PD1‐positive cells correlated with a shorter time to transformation.

Interestingly, among the 9 PD‐L1‐positive DLBCL cases, 5 had PD‐L1‐positive tumor cells only, another 3 had PD‐L1‐positive macrophages only, and just 1 case showed both types of cells to be PD‐L1‐positive. Among the 6 PD‐L1‐positive p‐PTLD cases, all had PD‐L1‐positive macrophages including 3 which also showed PD‐L1‐positive tumor cells. In the single PD‐L1‐positive case of non‐destructive PTLD only the macrophages were PD‐L1‐positive. This observation shows that although the rate of PD‐L1 expression is similar between m‐PTLD and p‐PTLD, the predominant type of PD‐L1‐positive cell is different with the macrophages almost always being involved in the less aggressive category of PTLD. Intriguingly, recent research has implied a significant role of non‐malignant tumor‐associated PD‐L1‐positive macrophages in, that is, Hodgkin's disease, with respect to prognosis, suggesting that with a higher amount of PD‐L1‐positive macrophages response to chemotherapy becomes poorer.^36^ Of the 4 p‐PTLD cases 3 showed, at least, persisting disease.

Numerous studies have tried to identify genetic predictors attributable to PD‐L1 expression in tumor cells.^27^, ^31^ To date, genetic alterations involving the PD‐L1 and PD‐L2 locus (9p24.1) as well as their induction via JAK2 signaling have been identified in a subset of PTLD.^37^ However, even in the latter studies the contribution of EBV was considered to be the main disease‐driving factor. There is long‐standing evidence that EBV core proteins, such as EBNA2, upregulate PD‐L1 to evade immune surveillance. However, in the context of lymphomagenesis various non‐genetic factors such as miR‐34 and others, seem to influence PD‐L1 expression, as well.^38^ In our study, apart from 3 of 29 PTLD samples analyzed, all showed EBV positivity on all tested occasions. Intriguingly, the highest PD‐L1 positivity of tumor cells was observed in 1 of 2 EBV‐negative DLBCL cases, indicating an alternative pathway of upregulation.

We acknowledge that our small series does not allow strong conclusions, but the inter‐ and intra‐individual correlation of PD‐L1 and PD1 expression with the PTLD category is conspicuous. The amount of PD‐L1‐ and PD1‐positive cells changed in the opposite way in sequential biopsies of the same individual correlating well with progression, persistence and regression of the WHO PTLD category. Accordingly, we observed less PD‐L1 expression in non‐destructive lesions and higher expression in p‐ and m‐PTLD. Since PTLD in childhood represents a therapeutic challenge when reduction of immunosuppression and standard immune and chemotherapy fail or are not possible, our results indicate a potential efficacy of checkpoint inhibitors for this group of patients, at least for p‐ and m‐PTLD. Therefore, co‐staining of PD‐L1 and PD‐1 is advisable in this group of patients and should be routinely implemented for immunohistochemical analysis of PTLD of childhood and adolescence. Nevertheless, as the use of checkpoint inhibitors in this very peculiar group of transplanted patients could be associated with the risk of auto‐/allo‐immune reactions, graft‐versus‐host disease, graft rejection, among others, a phase 1 and  2 trial in refractory PTLD or PTLD not tolerating standard therapies should be considered in order to assess safety and efficacy in a controlled setting.

## CONFLICT OF INTERESTS

The authors declare no competing financial interests.

## AUTHOR CONTRIBUTIONS

AA, AIS, ES, and ISK designed and planned the study; AIS, ES, CH, and AA wrote the manuscript. AIS, ES, and AF collected and analyzed the data. AIS and ISK were in charge of the histopathological analyses. All the other authors (ZS, TMS, WDH, IMB, AL, HP, GM) as well as AA, ES, and CH recruited, treated, and identified the patients. All authors read and approved the final version of the manuscript.
