# Supplementary material for: PD‐L1 and PD1 expression in post‐transplantation lymphoproliferative disease (PTLD) of childhood and adolescence: An inter‐ and intra‐individual descriptive study covering the whole spectrum of PTLD categories
Source: Cancer Med. 2019 Jul 3;8(10):4656–68. doi: 10.1002/cam4.2394 (PMC6712474; doi:10.1002/cam4.2394)
Supplement: Supplementary file 1 [file CAM4-8-4656-s001.docx]

**Supplemental table 1. Additional characteristics of the 21 patients with post-transplantation lymphoproliferative disease**

| **Pt. No.** | **Age at TX** | **Age at PTLD** | **Time btw. TX and PTLD** | **Diagnosis** | **Subtype of PTLD** | **Availability of samples** | **CD20 (%)** | **Ki-67 (%)** | ***c-MYC* rearrangement** |
| --- | --- | --- | --- | --- | --- | --- | --- | --- | --- |
|  |  |  |  |  |  | **for immunostaining** |  |  |  |
|  |  |  |  |  |  |  |  |  |  |
| 1 | 11,56 | 12,18 | 0,62 | m-PTLD | DLBCL | yes | + | 80 | negative |
| 2 | 11,94 | 12,11 | 0,17 | m-PTLD | DLBCL | yes | + (30%) | 80 | negative |
| 3 | 7,45 | 7,57 | 0,12 | m-PTLD | DLBCL | yes | + (20%) | 80 | negative |
| 4 | 0,64 | 11,69 | 11,05 | m-PTLD | DLBCL | yes | + | 70 | negative |
|  |  |  |  | m-PTLD | DLBCL | yes | + | 80 | n. d. |
| 5 | 12,55 | 21,88 | 9,34 | m-PTLD | DLBCL | yes | + | 95 | negative |
| 6 | 6,59 | 16,28 | 9,69 | m-PTLD | DLBCL | yes | + (30%) | 90 | negative |
| 7 | 5,67 | 6,19 | 0,52 | m-PTLD | DLBCL | yes | + | 80 | negative |
| 8 | 18,56 | 19,19 | 0,63 | m-PTLD | DLBCL | yes | + | 70 | negative |
| 9 | 16,65 | 17,27 | 0,62 | m-PTLD | DLBCL | yes | + | 70 | negative |
| 10 | 16,71 | 17,46 | 0,75 | m-PTLD | DLBCL | yes | + | 70 | negative |
| 11 | 14,06 | 16,63 | 2,56 | non-destructive PTLD | PH | yes | + | 20 |  |
|  |  |  |  | m-PTLD | PBL | no | - | 80 | n.d. |
| 12 | 10,13 | 21,81 | 11,68 | m-PTLD | PBL | yes | - | 100 | positive |
| 13 | 1,29 | 7,12 | 5,83 | m-PTLD | BL | yes | + | 95 | positive |
| 14 | 1,27 | 8,78 | 7,51 | m-PTLD | MALT lymphoma | yes | + | 10 | n.d. |
| 15 | 0,48 | 13,04 | 12,56 | p-PTLD |  | yes | + | 50 |  |
|  |  |  |  | p-PTLD |  | yes | + | 30 |  |
|  |  |  |  | m-PTLD | DLBCL | no | + | n.d. | n.d. |
| 16 | 8,21 | 8,37 | 0,16 | p-PTLD |  | yes | - | 40 |  |
| 17 | 1,48 | 2,52 | 1,04 | p-PTLD |  | yes | + | 20 |  |
|  |  |  |  | p-PTLD |  | yes | + | 20 |  |
|  |  |  |  | non-destructive PTLD | FFH | yes | + | 5 |  |
| 18 | 3,05 | 8,85 | 5,80 | p-PTLD |  | no | + | 30 |  |
|  |  |  |  | p-PTLD |  | yes | + | 10 |  |
|  |  |  |  | non-destructive PTLD | FFH | yes | + | 5 |  |
| 19 | 1,91 | 3,60 | 1,69 | non-destructive PTLD | IM | yes | + | n.d. |  |
|  |  |  |  | p-PTLD |  | yes | + | 30 |  |
| 20 | 1,42 | 1,86 | 0,44 | non-destructive PTLD | IM | yes | + | 30 |  |
| 21 | 1,13 | 2,92 | 1,79 | non-destructive PTLD | FFH and PH | yes | + | 5 |  |
|  |  |  |  | non-destructive PTLD | FFH and PH | yes | + | 5 |  |
|  |  |  |  | non-destructive PTLD | FFH and PH | yes | + | 10 |  |
|  |  |  |  |  |  |  |  |  |  |

**Abbreviations:** Pt. No., patient number; TX, transplantation; PTLD, post-transplantation lymphoproliferative disease; m-PTLD, monomorphic PTLD; p-PTLD, polymorphic PTLD; DLBCL, diffuse large B-cell lymphoma; PH, plasma cell hyperplasia; PBL, plasmablastic lymphoma; MALT, mucosa-associated lymphoid tissue; FFH, florid follicular hyperplasia; IM, infectious mononucleosis; n.a., not available; +, positive; -, negative; n.d., not done;
